# Supplementary material for: A Facile and Versatile Technique for Creating Antifibrotic Coatings on Biomedical Implants
Source: bioRxiv. 2026 Jun 9:2026.06.04.730237. Preprint. [Version 1] doi: 10.64898/2026.06.04.730237 (PMC13277944; doi:10.64898/2026.06.04.730237)
Supplement: Supplementary file 1 [file media-1.pdf]

A Facile and Versatile Technique for Creating Antifibrotic Coatings on Biomedical Implants

Yang Liu[1,2+], Connor Edvall[1,2+], Sudip Chakraborty[1,2], Achal Anand[1,2], Joy Agus[1,2] Suman Bose[1,2,3*]

[1] Department of Physiology and Biomedical Engineering, Mayo Clinic Arizona, Scottsdale, USA

[2] Department of Immunology, Mayo Clinic Arizona, Scottsdale, USA

[3] Mayo Clinic Comprehensive Cancer Center, Mayo Clinic Arizona, AZ, USA

[+]These authors contributed equally

*Corresponding author, Bose.Suman@mayo.edu

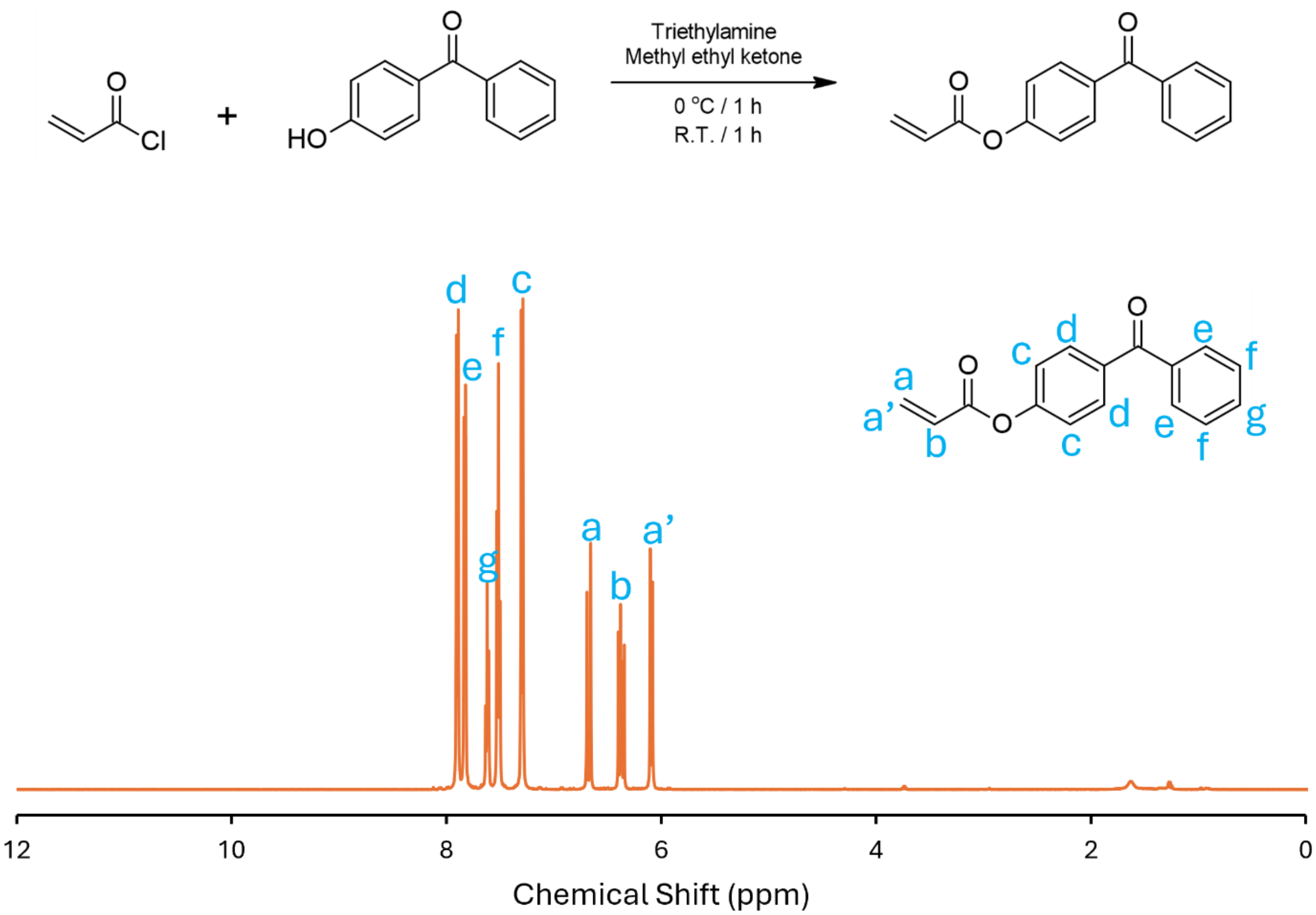

Figure S1. Synthesis scheme of BPA and its corresponding ^{1}H NMR spectrum.

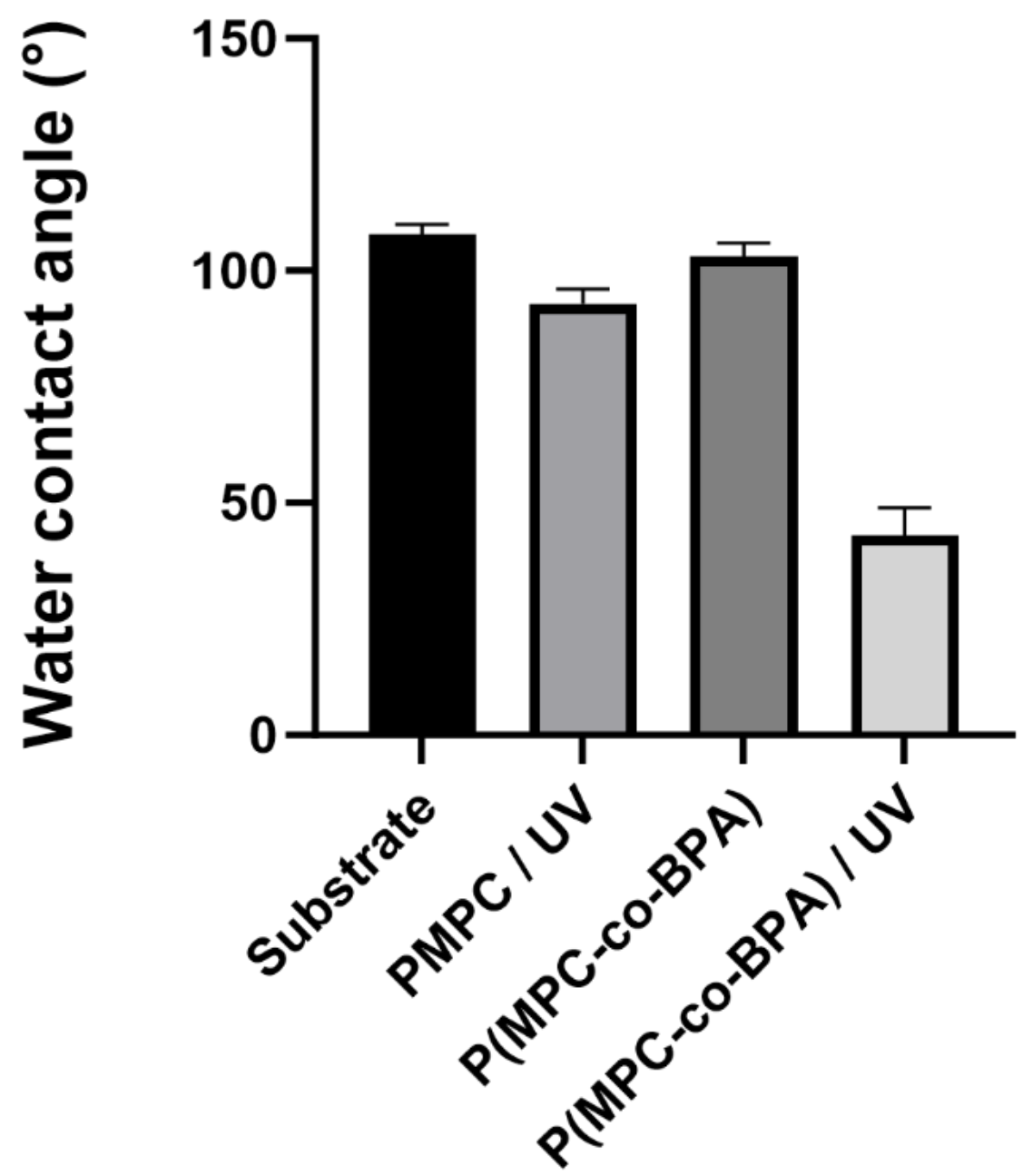

Figure S2. Water contact angles of polymer coatings in the presence or absence of BPA and UV Curing.

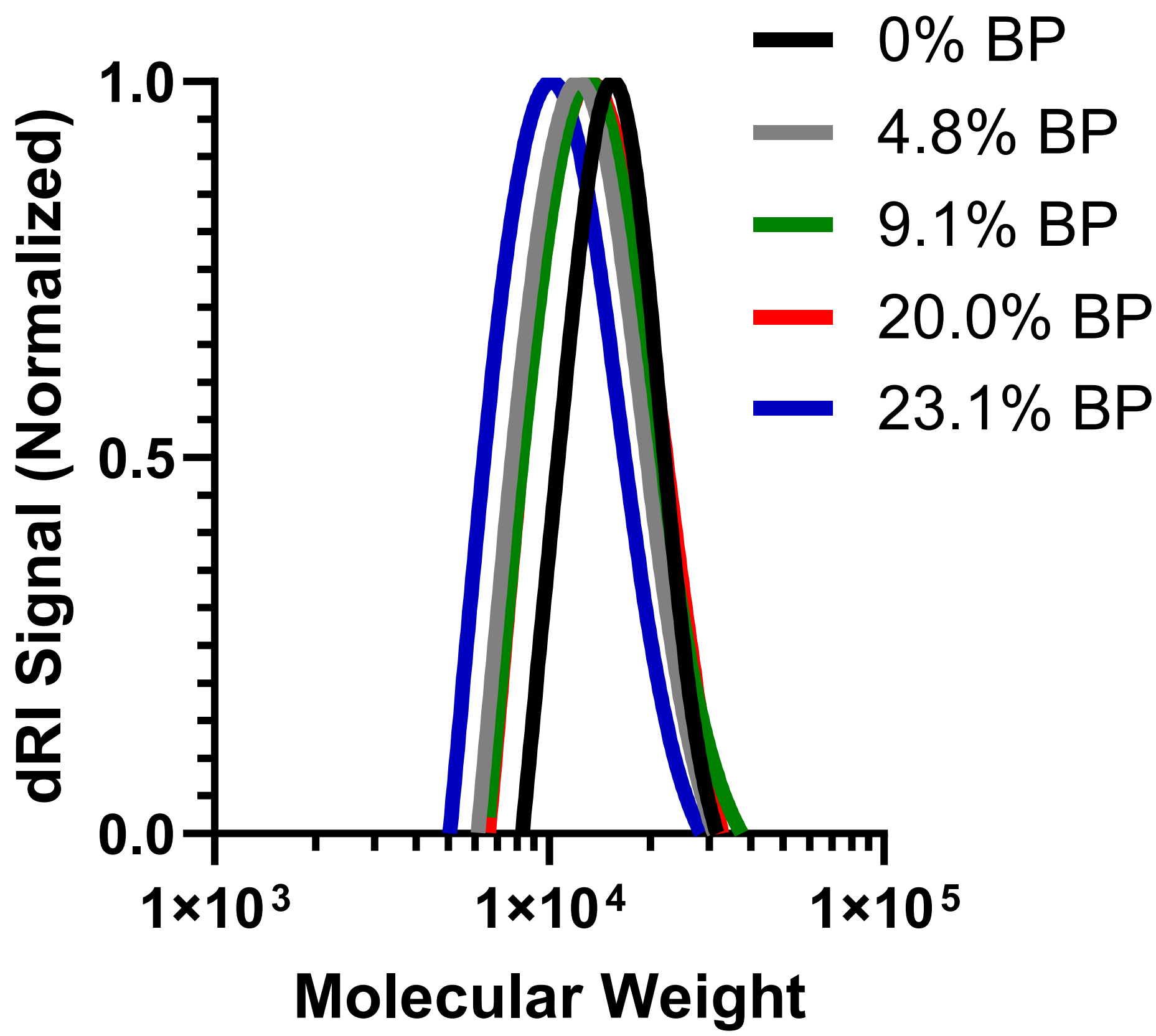

Figure S3. Distribution of varied MPC:BP polymer combination molecular weights confirmed via GPC.

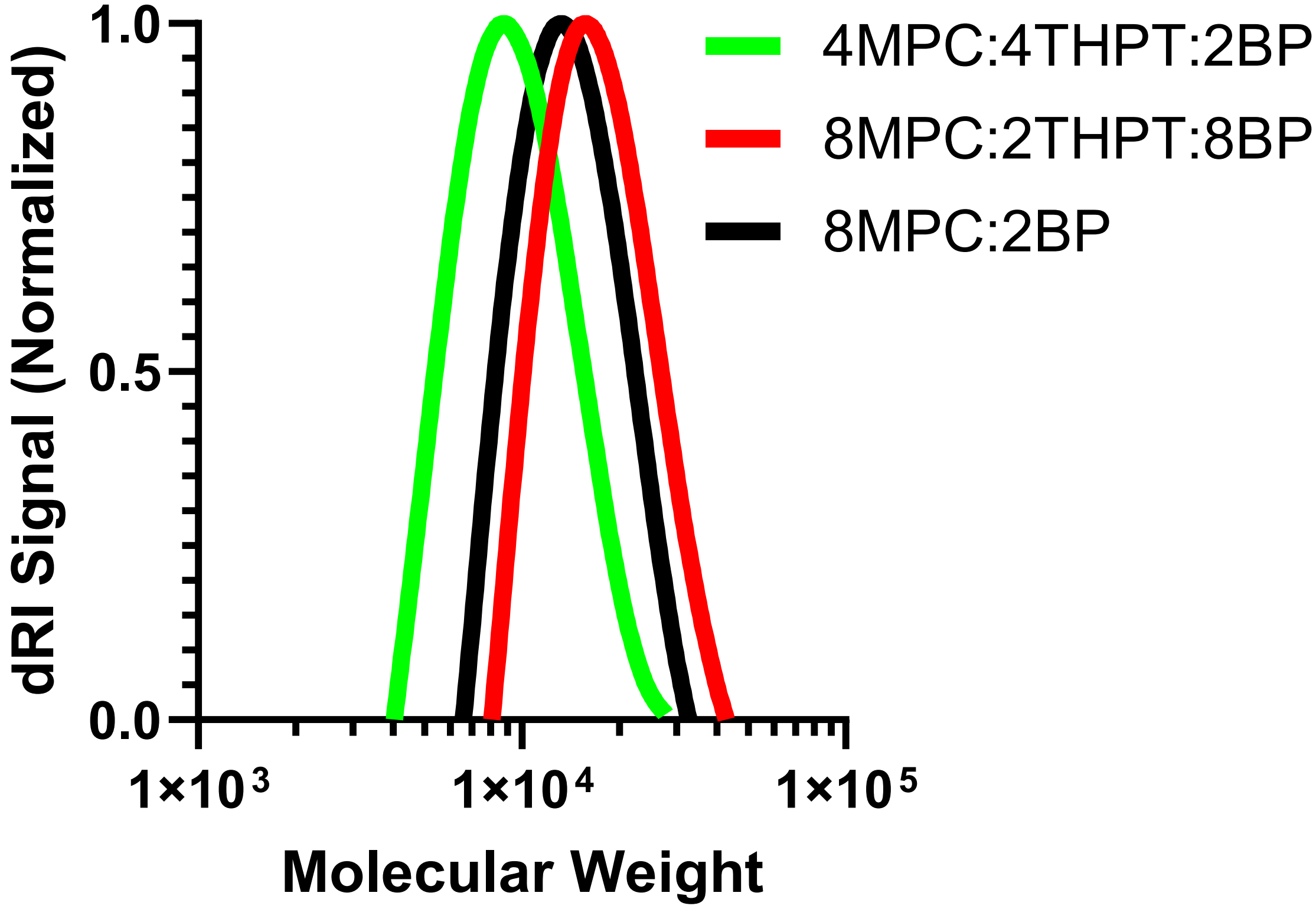

Figure S4. Distribution of varied MPC:THPT:BP polymer combination molecular weights confirmed via GPC.

Table S1. Molecular weight characteristics of copolymers prepared with different feed ratios. * indicates incompatibility with aqueous and organic phase GPC.

MPC:THPT:BP	0 : 8 : 2[a]	2 : 6 : 2[a]	4 : 4 : 2[b]	6 : 2 : 2[b]	8 : 0 : 2[b]
M_n	*	*	13600	15700	13100

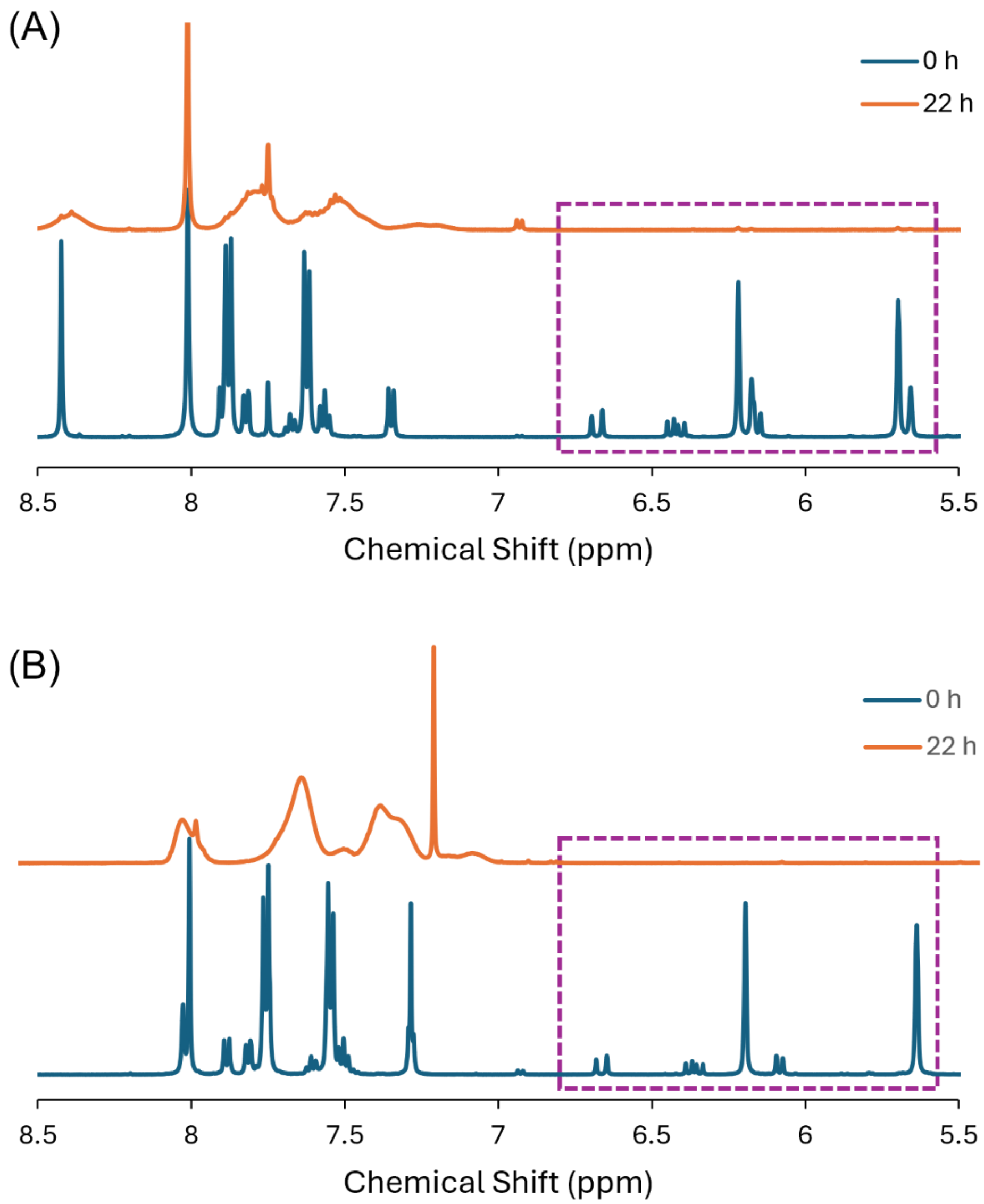

Figure S5. Monomer consumption of synthesis MPC : THPT : BPO = 2 : 6 : 2 (A) and MPC : THPT : BPO = 0 : 8 : 2 (B). The disappearance of double bond within 5.5 – 7.0 ppm indicating the consumption of monomers.

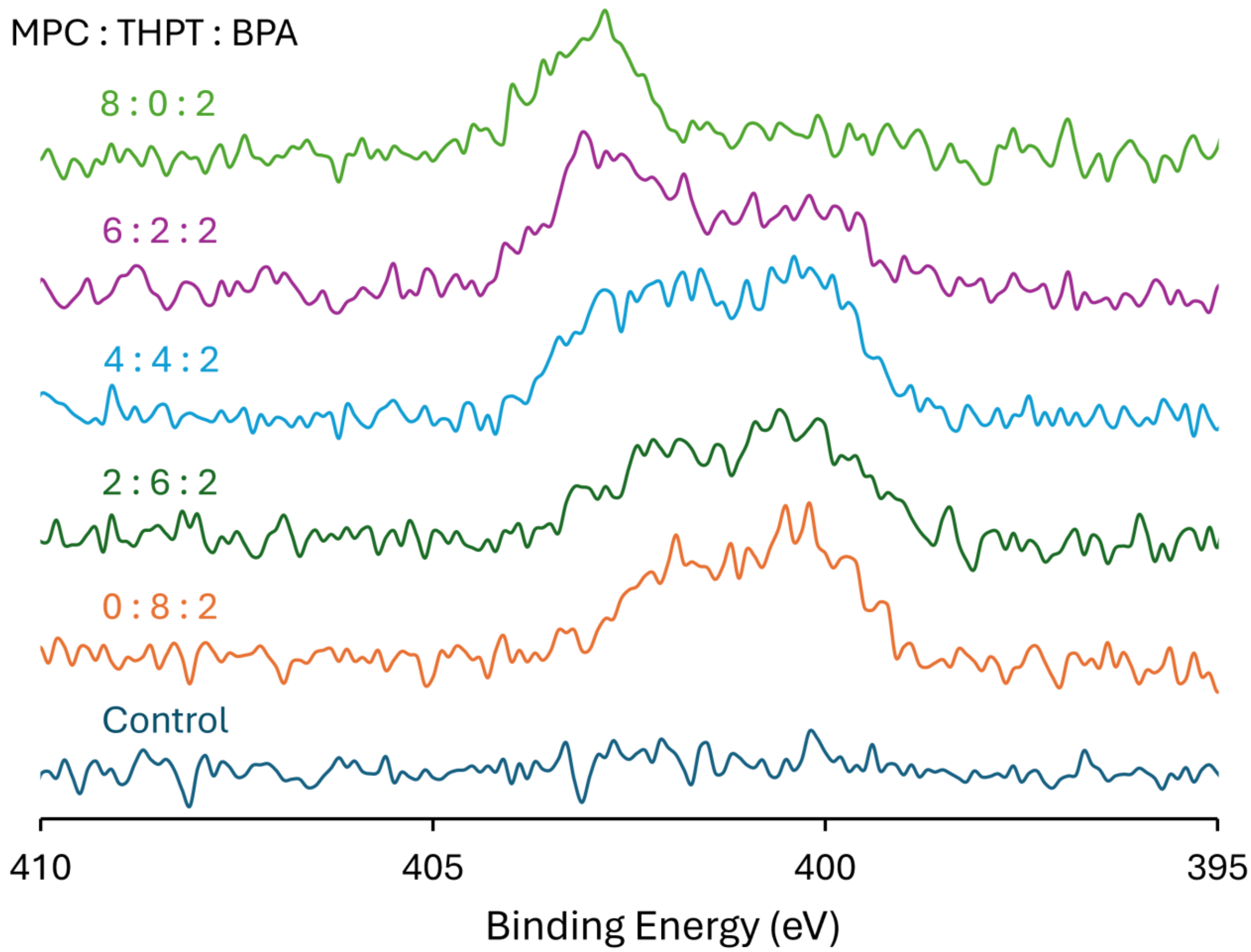

Figure S6. High resolution XPS spectra of polymer coatings prepared with different feed ratios.
